# Genetic Analysis for Fruit Phenolics Content, Flesh Color, and Browning Related Traits in Eggplant (*Solanum melongena*)

**DOI:** 10.3390/ijms20122990

**Published:** 2019-06-19

**Authors:** Prashant Kaushik

**Affiliations:** Instituto de Conservación y Mejora de la Agrodiversidad Valenciana, Universitat Politècnica de València, 46022 Valencia, Spain; prakau@doctor.upv.es; Tel.: +34-963-877-000

**Keywords:** chlorogenic acid, diallel, eggplant, GCA, SCA, SNP

## Abstract

Eggplant varieties rich in bioactive chlorogenic acid along with less browning are preferred by consumers. Therefore, genetics of fruit phenolics, fruit flesh colour, and browning related traits were studied in the genotypes of eggplant, comprising of nine cultivated varieties and one accession of eggplant‘s primary genepool wild relative *Solanum insanum* (INS2). These accessions were genotyped based on the 7335 polymorphic single-nucleotide polymorphisms (SNP) markers. After that, genotypes were crossed in half diallel fashion to produce 45 hybrids. The INS2 displayed the highest values for the total phenolics and chlorogenic acid content (CGA). For all of the biochemical traits studied, significant values of general and specific combining ability (GCA and SCA) effects were determined. The baker ratio estimates were high (>0.75) for all of the traits. Highly significant and positive heterosis (%) was determined for the dry matter, total phenolics, CGA, and area (%) of CGA content. The phenolics content of the fruit (total phenolics and CGA) was not significantly correlated with flesh colour and browning related traits. However, when the path coefficient analysis was performed considering the CGA as a dependent variable, it was determined that the flesh colour related traits most considerably affected the CGA. The genetic distance showed a diminutive correlation with the hybrid means, heterosis, and SCA values. Overall, this study provides important information regarding the underlying genetics of important biochemical traits of eggplant fruit.

## 1. Introduction

Eggplant (*Solanum melongena* L.) is the third most consumed fruit of the family Solanaceae [1,2]. Eggplant has high beneficial effects on human health due to its high content of phenolic acids [3,4,5]. These phenolic acids are important for their various health promoting effects such as protection against chronic diseases such as cancer and arthritis [6]. Among the different types of phenolic acids identified in eggplant, chlorogenic acid is the most frequent type and it makes up to 90% of the total phenolic acids in eggplant [5,7]. The phenolic acid content in eggplant flesh varies among cultivars, and also, the wild relatives of eggplant generally have higher diversity and concentrations of phenolic acid content than the modern cultivated varieties [8,9].

Various reports suggest that increasing the phenolic acid content in the fruit flesh increases the susceptibility of eggplant flesh to browning [10,11]. In this way, previous studies have pointed out that chlorogenic acid content moderately influences the fruit flesh browning in an eggplant [12]. In order to develop modern eggplant cultivars with a higher content of phenolics, several kinds of genetic materials have been screened and a significant amount of variation in phenolic acid content has been observed in the cultivated varieties, wild species, and also interspecific hybrids [8,9,13]. Recently, we studied the diversity of phenolic acid content in cultivated eggplant and its wild relatives from all the primary, secondary, and tertiary genepools [8,14].

Diallel-based genetic studies provide information to determine the variations of the trait in question and identify parents and cross combinations likely to produce better hybrids [15,16]. The half-diallel mating design, which includes one-way direct crosses and their parents [17,18], provides valuable information regarding the combining abilities of parents, which are the critical predictors of the breeding value of hybrids. In this way, general combining ability (GCA) indicates additive gene action, while the specific combining ability (SCA) points towards the nonadditive gene action, which can be caused by dominance, epistasis, and overdominance effect in controlling the trait in question [19].

The genome eggplant sequence is already available [20], and several studies have been conducted using molecular markers from random amplification of polymorphic DNA (RAPDs) to more recent ones with SNPs [20,21]. Several studies have used these molecular markers to estimate the genetic distances among parents and evaluate their value to predict the performance of hybrids [22,23,24,25]. However, in eggplant, there is limited knowledge about the use of molecular markers to predict hybrid performance [23], and to our knowledge no studies concerning the potential of molecular markers for predicting the fruit phenolic content, fruit colour, and browning of hybrids. Moreover, for insights regarding the contributions of all independent variables on a dependent variable, path coefficient analysis is considered to be a highly efficient method and has not been applied to biochemical traits such as chlorogenic acid content in eggplant [26]. Therefore, the present investigation was undertaken to provide information on the genetics and inheritance of phenolic acid content, fruit flesh colour, and browning in eggplant. In our study, we estimate combining abilities (GCA and SCA), heritabilities, and determine the usefulness of SNPs based genetic distances for predicting the performance of hybrids for these traits.

## 2. Results

### 2.1. Variation in Parents and Hybrids

The average values (means) of the parental genotypes and hybrids were similar for most of the traits studied (Table S1). Interestingly, the coefficient of variation was in the parental genotypes as compared with the hybrids (Table 1). Furthermore, the coefficients of variation were larger in values in the parents than their hybrids (Table 1).

The estimates of the mean sum of squares (ANOVA) for the general combining ability (GCA) of parents, and the specific combining ability (SCA) of the hybrids were highly significant (*p* ≤ 0.01) (Table 2). In general, the values of the GCA effects were higher than the values of the SCA effects (Table 2). The predominance of additive gene action was noticed based on the Baker ratio (>0.75) for all of the traits studied (Table 2). The estimates of broad-sense heritability (≥0.50) were larger as compared with those for narrow-sense heritability (≤0.50) (Table 2). The CGA content was determined with the lowest values for both narrow-sense (0.02) and broad-sense (0.23) heritability (Table 2). Dry matter, total phenolics, chlorogenic acid content (CGA), area%, L*0, a*0, b*0, degree of whiteness (DW_0_), polyphenol oxidase activity (PPO), and fruit flesh degree of browning (DB) showed low (≤0.30) narrow-sense heritability. Interestingly, all traits, except CGA (0.23), exhibited a broad-sense heritability value above 0.5 (Table 2).

### 2.2. GCA and SCA Effects

The GCA effects for dry matter ranged from −0.87 (MEL1) to 1.29 (INS2), and for the phenolics from −2.23 (A0416) to 2.37 (INS2) (Table 3). For CGA, the general combining ability estimates were nonsignificant for all the parents except for ASI-S-1 (−0.27) and INS 2 (0.39) (Table 3). The general combining ability effects for area% CGA ranged from −8.60 (MM1597) to 3.60 (IVIA-371) (Table 3). The general combining ability estimates for L* ranged from −3.14 (INS 2) to 3.00 (MEL 5), while they ranged from −0.91 (DH 621) to 1.22 (A0416) and from −2.90 (MEL 1) to 3.30 (MM1597) for a*_0_ and b*_0,_ respectively (Table 3). The GCA effect for PPO activity ranged from −0.50 (IVIA-371) to 1.30 (INS 2). The GCA effect for the DB and the fruit flesh colour difference (CD) ranged from −0.85 (MEL 5) to 3.25 (INS 2) and −1.50 (MEL 5) to 4.35 (INS 2), respectively (Table 3).

The SCA effects are presented in Table 4. For the dry matter content, the highest positive SCA values of 2.64, 2.47, and 2.28 were observed in the cross combinations IVIA-371 × MEL5, MM 1597 × H15, and DH 621 × IVIA-371, respectively (Table 4). For phenolics, significant SCA effects were recorded for the cross combinations H15 × IVIA-371 (5.97), AN-S-26 × ASI-S-1 (4.90), and DH 621 × MEL 1 (3.37), respectively (Table 4). The highly significant positive SCA effects for CGA were recorded for the cross combinations H15 × IVIA-371 (1.08) and IVIA-371 × INS2 (0.65) (Table 4). The following cross combinations: IVIA-371 × INS2 (13.44), IVIA-371 × MEL 5 (12.25), and DH 621 × ASI-S-1 (11.36) exhibited a significant SCA effect for the area% under the curve (Table 4).

The positive and high SCA effects of 4.50, 4.30, and 3.40 for the L*_0_ in the cross combinations IVIA-371 × INS2, AN-S-26 × H15, and DH 621 × INS2, respectively (Table 4). The significant and positive SCA effects for b*_0_ were recorded in the cross combinations DH 621 × MEL 5 (7.42), A0416 × MEL 5 (4.82), and DH 621 × IVIA-371 (3.97), respectively (Table 4). Likewise, the cross combinations with positive and significant SCA effects were A0416 × INS2 (7.52), DH 621 × MEL 5 (5.62), and ASI-S-1 × INS2 (5.54), respectively (Table 4). The negative (SCA) effects were preferred for the selection of the varieties with low PPO activity, DB, and CD and they were identified as MM 1597 × INS2 (−2.81), IVIA-371 × INS2 (−1.81), and A0416 × INS2 (−1.70), respectively (Table 4). Whereas for the DB and the CD the cross combinations identified with highly significant negative SCA effects were IVIA-371 × INS2 (−4.76, −6.82), DH 621 × INS2 (−3.13, −5.16), and ASI-S-1 × INS2 (−2.78, −2.60), respectively (Table 4).

### 2.3. Heterosis

**Figure 1:**
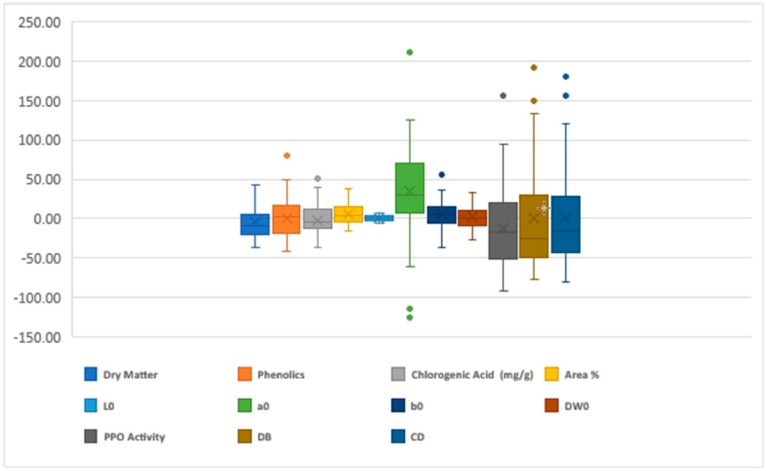
The boxplots (median and interquartile range) representing the mid-parent heterosis (%).

Highly significant heterosis was measured for all the characters studied (Figure 1). The lowest fluctuation for the heterosis range was noticed for the L*_0_ (6.97) while the highest fluctuation was present for the a*_0_ (211.28) (Figure 1). The highly significant positive heterosis measured for the dry matter, total phenolics, CGA, and area were 43.30, 79.48, 50.77, and 38.47, respectively. Whereas, the desired highly significant negative heterosis was noticed for PPO (91.67), DB (−63.70), and CD (−80.66), respectively (Figure 1).

### 2.4. Correlations and Path Analysis

Twenty-one out of a total of fifty-five correlations were significant at *p* < 0.05. Three of these correlations presented high absolute values (~0.90); two of these were positive correlations (between DB and CD, and between b*_0_ and DW_0_), while the other one was negative (between L*_0_ and DW_0_) (Table 5). Dry matter was positively correlated with DB and CD (Table 5). Total phenolics and GCA were not correlated with any other trait. However, when considering the area percentage of chlorogenic acid chromatogram, it was found to be positively correlated to L*_0_ and negatively correlated to b*_0_, DW_0_, PPO activity, and CD (Table 5). A moderately positive correlation of PPO activity was noticed with DB and CD (Table 5).

**Figure 2:**
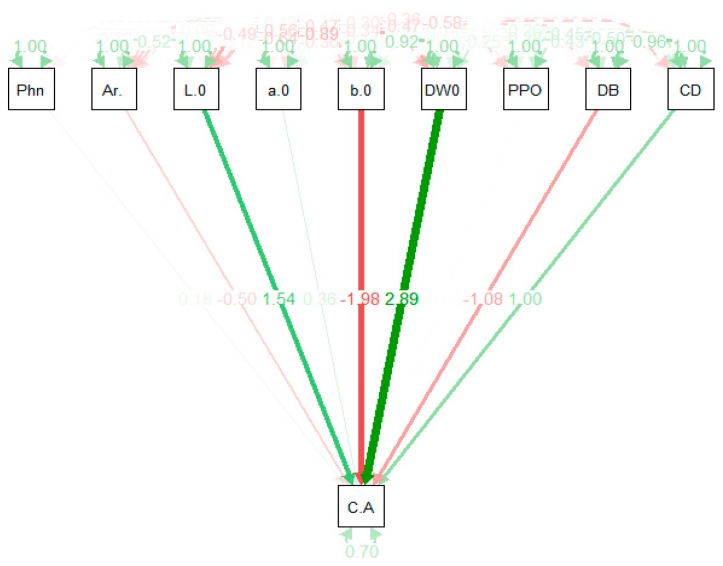
Path coefficient analysis considering chlorogenic acid content as the dependent variable on all other remaining traits.

Simple correlations between traits do not provide very reliable information regarding the components that resulted in this kind of relationship. The path coefficient analysis technique provides information regarding the independent variables and the way they affect a dependent trait (directly or indirectly). The standardized effect of both latent and observed variables is provided in Figure 2. The largest positive effect was exhibited by DW_0_ (2.89) followed by L*_0_ (1.54), and the remaining chromameter parameter b*_0_ (−1.98) showed a negative effect (Figure 2). Whereas, total phenolics content showed no effect on the chlorogenic acid content (Figure 2).

### 2.5. Genetic Distances and Correlation with Hybrid Performance and Genetic Parameters

The cluster analysis results showed that the eggplant wild relative, INS2, was clustered with MEL1 (from Africa) and MEL5 (from Asia), whereas, the remaining genotypes were clustered together (Figure S1). Furthermore, the entanglement coefficient was 0.29, suggesting a good overall alignment based on SNPs and eleven traits (Figure S1). Among the cultivated accessions, the maximum genetic distance (GD) was observed between the A0416 and MEL1 (Table 6). Whereas, genotype DH621 was determined to be very similar to genotypes AN-S-26, H15, and IVIA-371. For all 45 hybrids, the genetic distance was significant for four traits out of the total of 11. The traits that significantly correlated with the genetic distance were a*_0,_ b*_0,_ and CD (Table 6). Interestingly, for the heterosis and SCA effects, only PPO activity was found to be negatively correlated with the genetic distance (Table 6). When excluding the hybrids with *S. insanum,* the significant r values were determined for all four flesh colour related parameters L*_0,_ a*_0,_ b*_0,_ and DW_0_. Whereas, trait heterosis was not significantly correlated with the genetic distance for any of the traits. The L*_0_ was the only trait where the SCA effects were correlated with the genetic distance (Table 6).

## 3. Discussion

Eggplant is among the fruits with the highest phenolic compound content [27]. However, the oxidation of phenolic acids produces brown compounds which may impede the development of commercially successful eggplant varieties [12]. Nevertheless, knowing the association among the descriptors is helpful for efficient breeding. Generally, the identification of a suitable donor parent, evaluating the genetic variation and diversity is important for successful breeding [28,29,30].

Generally, in the case of self-pollinated crops like eggplant, the alleles are mostly fixed, and genetic variation is limited among the popularly cultivated varieties [31,32]. Under such circumstances, the underexploited variability present in the different genepools on the farm of landraces and crop wild relatives is highly useful which can donate valuable genes for the improvement of the cultivated varieties [6,27]. In our study, we used the nine accessions that differed in shape and sizes, along with one accession of *S. insanum*. Overall, the mean sum of squares due to GCAs were higher than otherwise due to SCA and this generally favours selection breeding methods. Previously, the selection breeding methods were extensively used for the improvement of biochemical traits [33,34].

The diallel mating design, excluding reciprocals, is a robust and manageable design for a better understanding of combining abilities and gene actions of the genes governing the important traits of eggplant [23,35]. This information on combining abilities and gene actions is of interest to breeders in order to devise a proper breeding strategy that involves suitable parents [36]. Here, we found that only the wild accessions, i.e., INS2 had highly significant GCA effects for the traits except for the fruit color related trait. Moreover, INS2 was positively significant for the flesh browning related traits where the direction of acceptability and selection were negative. We determined that INS2 was highly significant for the total phenolics and CGA content. *S. insanum* has an immense potential to contribute several favourable genes to modern eggplant cultivars [37].

However, in the past, wild relatives have contributed to the improvement of several traits in other solanaceous fruits or vegetables such as tomato and potato, respectively [38]. In addition, recently we have found that the wild relatives are sometimes three times higher in value for the important total phenolics and GCA content [8]. The significant SCA effects were scattered among the several cross combinations. For phenolics, the significant SCA effects were recorded in the cross combinations AN-S-26 × ASI-S-1, and DH 621 × MEL 1. Surprisingly, significantly positive SCA effects for CGA were recorded for the different cross combinations H15 × IVIA-371 and IVIA-371 × INS2. This points out the presence of several kinds of phenolic acids in eggplant flesh that might also express more with distant cross combinations using wild relatives [9].

Interestingly, phenolics and chlorogenic acid contents were not correlated with each other and also were not correlated with any other trait studied, i.e., with DW_0_, PPO activity, and DB. However, the area percentage of GCA was negatively correlated with all browning and colour related traits (except L*_0_). These results are in agreement with our previous findings. Earlier it was also shown that higher phenolics are not associated with the fruit browning [8]. To determine the indirect selection criterion for chlorogenic acid content via path coefficient analysis, traits with positive direct effects (L*_0_ and DW_0_) as well as with positive correlation values can be considered [26]. The wild relative accession, INS2, was clustered with MEL1 and MEL5. The reason for this clustering could be the similarity of the cultivated varieties in the primary centre of origin of the eggplant (Asia and Africa) with the primary genepool species *S. insanum*. Moreover, the *S. insanum* is commonly cultivated in Asia and Africa along with other more elite varieties [2,37].

Crossing a line into a different cross combination gives information about that line in all its cross combinations. The cross with its specific value is a result of the sum of GCA of two lines used in that particular cross combination. The SCA estimates are useful for finding the particular cross combinations in the farm of heterosis for the highest expression of a trait. However, the preferred parents are those where one parent has a high GCA while the overall cross combination is a high SCA value. Additive gene action for those traits demonstrates that it is better to use it and perform an efficient selection. This information on the quantitative genetics of eggplant is used for inference decisions on parental choice when breeding for various morphological traits [39]. Therefore, the present studies were carried out to understand the nature of gene action governing the inheritance of important morphological traits of eggplant, as well as, to identify and develop a deeper understanding of the combining abilities of parents and their hybrids and to correlate this information with their genetic distance obtained by using SNPs.

## 4. Materials and Methods

### 4.1. Plant Material, Growing Conditions, and Sample Preparation

Nine eggplant cultivars and one accession of the eggplant primary genepool wild species *S*. *insanum* (INS2) were used for this study. The eggplant cultivars were previously found to be morphologically diverse, and their main characteristics were described in a study by Kaushik et al. [23]. These 10 genotypes were crossed in the diallel mating design without reciprocals to produce 45 F_1_ hybrids. All the parental plants and hybrids were grown under the open field situation in a plot located at the Universitat Politècnica de València (coordinates at: 39°28′55″ N, 0°22′11″ W; altitude 7 m a.s.l.). Three replications consisting of three plants were distributed according to a randomized complete block design. Plants were watered employing drip irrigation, and fertigation was provided by distributing 80 g·plant^−1^ of a 10 N, 2.2 P, 24.9 K plus micronutrients fertilizer (Hakaphos Naranja, Compo Agricultura, Barcelona, Spain) throughout the cultivation period using the irrigation system. At the appropriate age, plants were trained on bamboo canes. Weeds were manually removed and no phytosanitary measures were needed.

Samples from each replication consisted of five fruits, which were picked at a commercially ripe stage (physiologically immature) for the characterization of phenolics, fruit colour, and browning. Fruits were opened transversally, and half of the fruit was snap frozen using liquid nitrogen that was kept at −80 °C untill further use, while the other half of the fruit was used for measuring the flesh browning.

### 4.2. Characterization of Fruit

Fruit flesh browning was measured using a CR-300 chromameter (Minolta, Osaka, Japan) at the midpoint position (the centre of the fruit) in each of the five fruits that constituted one sample. The values for CIELAB colour parameters L*, a*, b* were measured immediately after the fruit was cut (L*_0_, a*_0_, b*_0_), also, the fruit flesh colour was measured as the distance to DW_0_. New measurements of L*, a*, and b* parameters were taken after 10 min (L*_10_, a*_10_, b*_10_). These values were processed to estimate the DB and CD using the formulas as CD = [(L*_10_ − L*_0_)^2^ + (a*_10_ − a*_0_)^2^(b*_10_ − b*_0_)^2^]^0.5^ defined in detail by Prohens et al. [13].

The percentage of change in weight before and after the lyophilization process was used as the measure of dry matter content. The Folin–Ciocalteu spectrophotometric method was used to measure the total phenolics (mg/g dw) of the eggplant flesh as defined in detail in [40]. The total phenolics content was quantified using chlorogenic acid as the standard for comparing the spectra at 750 nm with a spectrophotometer (Jenway, Essex, UK). The determination of CGA content was done with the help of high-performance liquid chromatography (HPLC) on a 1220 Infinity LC System (Agilent Technologies, Santa Clara, CA, USA). The calculations were performed by the OpenLAB CDS ChemStation Edition software package (Agilent Technologies) according to the manufacturer’s instructions [41]. The percentage of peak area for chlorogenic acid was determined using the chlorogenic acid peak area and a total peak area of other phenolic acids (mainly hydroxycinnamic acid conjugates). The polyphenol oxidase activity was determined based on the protocol defined in [8]. Briefly, a lyophilized sample of 0.1 g was homogenized with 4 mL of 0.1 M sodium phosphate buffer (pH 6.0). This mix was centrifuged for 15 min at 12,000 rpm (4 °C). The supernatant was collected and further diluted with a buffer extraction solution (5-fold). The PPO evaluation was determined with a total volume of 2 mL comprising of 50 µL of diluted supernatant (enzyme extract), 150 µL of 0.1 M chlorogenic acid (dissolved in 50% methanol), and 1.8 mL of 0.1 M sodium phosphate buffer (pH 6.0). The reaction activity was determined as the increase in the absorbance at 420 nm using a nanodrop ND-1000 spectrophotometer (Nanodrop Technologies, Montchanin, DE, USA). Furthermore, the unit change in enzyme activity was calculated as the increase in 0.1 absorbance unit per minute per milligram of dry weight.

### 4.3. Data Analysis

For each trait measured, the mean and range were calculated for the parental (*n* = 10) and hybrid (*n* = 45) groups. The mean values of parents and their hybrid combinations were compared with *t*-tests to detect differences among the two groups. The significance of differences among the group means was evaluated at *p* < 0.05 using the Statgraphics Centurion XVI software (StatPoint Technologies, Warrenton, VA, USA). Path coefficient analysis was performed by considering chlorogenic acid content as the dependent variable, using the software package Lavaan in R environment [42].

The diallel analysis was performed based on Griffing’s Method 2 (parents and F1 hybrids) and Model 1 (fixed effects) [17]. These calculations were done using the AGD-R (Analysis of Genetic Designs with R) software package [43]. The Baker ratio was estimated as GCA/SCA = 2 × s2GCA / (2 × s2GCA) + s2SGA [18]. The relative SCA values of individual hybrids were expressed as a percentage (%) over the average of the trait. The Statgraphics Centurion XVI software was used for the estiamtion of pairwise Pearson linear coefficients of correlation (r). The mid-parent heterosis of F1 (Het, %) was calculated using the formula Het = 100 × (F1 − MP)/MP, where, F1 = hybrid mean and MP = mean of the parents.

### 4.4. Genetic Distance and Its Correlation

Genotypic data was obtained for the ten accessions used in the study following the RAD sequencing approach used in previous studies [23,44]. In total 7335 polymorphic SNPs were used to determine genetic distances between the 10 parents used in our study. The TASSEL software version 5.0 Standalone was used to determine the genetic distances based on the identity-by-state (IBS) genetic distance (GD) as GD = 1 − IBS [45]. The genetic distance of parents of individual hybrids was further used to determine the Pearson linear correlations between the GD and hybrid trait values, heterosis, and SCA. The unweighted pair group method with arithmetic mean (UPGMA) was used to relate and visualize the relationships among the genotypes based on missing/detected SNPs and heterozygous/homozygous SNPs of the RAD sequenced file data [23]. Similarly, the UPGA distance-based dendrogram was made based on the eleven biochemical traits. Thereafter, a comparison of dendrograms was performed using both the tanglegram algorithm and the R package dendextend [46].

## Figures and Tables

**Table 1:** Mean and range of variation for all the biochemical traits studied.

Descriptors	Parents	Hybrids	*p*-Value
**Dry Matter**	10.07	9.41	0.2613
Range	(7.43 to 14.10)	(6.95 to 12.82)	
*CV%	22.50	15.81	
**Total Phenolics**	11.02	10.95	0.9458
Range	(7.58 to 15.97)	(5.81 to 17.47)	
CV%	25.95	27.65	
**Chlorogenic Acid (mg/g)**	2.88	2.81	0.5249
Range	(2.08 to 4.26)	(1.82 to 3.39)	
CV%	15.20	15.21	
**Area%**	69.78	72.86	0.2870
Range	(45.25 to 83.14)	(60.15 to 88.14)	
CV%	17.07	9.87	
L*_0_	81.02	81.57	0.6430
Range	(72.77 to 88.55)	(73.27 to 86.25)	
CV%	6.38	3.50	
a*_0_	2.33	3.04	0.1717
Range	(−4.31 to 0.08)	(−6.17 to 0.33)	
CV%	57.44	48.94	
b*_0_	17.40	18.25	0.5634
Range	(10.21 to 23.01)	(8.97 to 25.54)	
CV%	26.08	22.55	
**DW_0_**	26.12	26.39	0.8761
Range	(16.06 to 34.86)	(19.51 to 34.85)	
CV%	25.28	16.80	
**PPO**	2.75	2.03	0.1151
Range	(1.20 to 8.13)	(0.66 to 4.33)	
CV%	76.95	50.96	
**DB**	3.72	3.41	0.7554
Range	(1.48 to 9.35)	(0.76 to 16.45)	
CV%	70.97	82.80	
**CD**	6.14	5.38	0.5624
Range	(1.82 to 16.80)	(1.88 to 20.90)	
CV%	78.80	63.53	

*, coefficient of variation (CV%) estimated as the ratio of the standard deviation to the mean (average) × 100.

**Table 2:** Mean squares for block, genotypes, general combining ability (GCA), specific combining ability (SCA), Baker ratio, and narrow- and broad-sense heritabilities for the ANOVA for the fruit traits evaluated.

Descriptors	Block	Genotypes	GCA	SCA	Error	Baker Ratio	Narrow Heritability	Broad Heritability
d.f.	2	54	9	45	108			
Dry matter	2.21	8.17 ***	15.77 ***	6.65 ***	1.81	0.83	0.18	0.57
Total phenolics	23.83 *	26.48 ***	67.13 ***	18.35 ***	7.31	0.88	0.23	0.50
CGA	0.29	0.63 ***	0.94 ***	0.57 ***	0.30	0.77	0.09	0.23
Area%	25.32	201.58 ***	527.42 ***	136.41 ***	30.48	0.89	0.30	0.67
L*_0_	41.77 ***	33.40 ***	116.03 ***	16.88 ***	5.63	0.93	0.40	0.64
a*_0_	4.01	6.54 ***	20.45 ***	3.76 ***	1.60	0.92	0.31	0.53
b*_0_	32.89 ***	52.00 ***	205.52 ***	21.30 ***	4.56	0.95	0.52	0.79
DW_0_	69.38 ***	69.81 ***	268.83 ***	30.01 ***	8.08	0.95	0.48	0.73
PPO	0.09	5.09 ***	11.32 ***	3.85 ***	0.81	0.85	0.24	0.66
DB	0.60	23.08 ***	52.35 ***	17.22 ***	2.60	0.86	0.27	0.75
CD	3.23	40.55 ***	106.22 ***	27.41 ***	4.61	0.89	0.32	0.74

***, **, * indicate significant at *p* < 0.001, *p* < 0.01, or *p* < 0.05, respectively.

**Table 3:** General combining ability (GCA) estimates of the parents (*n* = 10).

Descriptors	MM 1597	DH 621	AN-S-26	HI5	A0416	IVIA-371	ASI-S-1	MEL 1	MEL 5	INS2
Dry Matter	−0.26	−0.40	0.37	0.88 ***	−0.26 ^ns^	−0.49 *	0.01	−0.87 ***	−0.27	1.29 ***
Total Phenolics	0.69	−1.10 *	−0.81	0.55	−2.23 ***	1.07 *	1.05 *	−0.60	−1.00 *	2.37 ***
CGA	−0.02	−0.02	−0.06	−0.02	−0.03	−0.02	−0.28 **	0.03 ^ns^	0.04	0.39 ***
Area%	−8.60 ***	1.23	−1.11	1.55	1.98 *	3.59 *	1.64	3.60 ***	0.62	−4.48 ***
L*_0_	−2.06 ***	−0.92 *	0.62	−0.33	−0.42	1.10 *	0.42	1.73 ***	3.00 ***	−3.14 ***
a*_0_	−0.83 ***	−0.91 ***	−0.43	−0.78 ***	1.22 ***	0.78 ***	−0.11	0.65 **	−0.10 ^ns^	0.51 *
b*_0_	3.30 ***	2.97 ***	0.17	1.78 ***	−0.82 *	−2.65 ***	1.56 ***	−2.89 ***	−3.15 ***	−0.27
DW_0_	3.86 ***	2.76 ***	−0.33	1.50 **	−0.32	−2.69 ***	0.76	−3.31 ***	−4.35 ***	2.13 ***
PPO	−0.23	−0.32 *	0.35 *	0.01	0.47 **	−0.50 **	−0.44 **	−0.22	−0.42 *	1.30 ***
DB	−0.40	−0.18	−0.57 *	0.18	0.38	−0.61 *	−0.74 *	−0.46	−0.85 **	3.25 ***
CD	−0.50	0.56	−0.66	0.75 *	0.33	−1.16 **	−1.00 *	−1.18 **	−1.50 ***	4.35 ***

***, **, * indicate significant at *p* < 0.001, *p* < 0.01, or *p* < 0.05, respectively.

**Table 4:** Specific combining ability (SCA) estimates of the hybrids (*n* = 45).

Hybrids	Dry Matter	Total Phenolics	CGA	Area%	L*_0_	a*_0_	b*_0_	DW_0_	PPO	DB	CD
MM 1597 × DH 621	−1.41 *	−0.38	0.47	2.11	−0.84	0.02	1.17	1.40	1.31 **	−1.20	−1.75
MM 1597 × AN-S-26	−2.14 ***	1.05	0.36	7.89 **	−0.22	0.90	2.51 *	1.83	−0.15	0.50	2.30 *
MM 1597 × H15	2.47 ***	−1.18	−0.58	−5.56	0.90	−0.42	0.70	0.02	0.90	−0.41	−0.56
MM 1597 × A0416	0.76	0.85	−0.23	5.17	2.22	−1.65 *	−3.66 ***	−3.93 **	−0.40	0.12	−0.20
MM 1597 × IVIA-371	0.18	1.12	0.06	3.11	1.51	−1.16	−2.07	−2.30	−0.50	−1.28	−1.38
MM 1597 × ASI-S-1	−0.55	−2.15	−0.04	−3.61	1.14	0.50	−1.14	−1.73	−0.20	−1.67	−2.61 *
MM 1597×MEL 1	−1.04	−0.74	−0.11	8.44 **	2.54 *	−0.60	−0.42	−2.26	−0.04	−1.44	−2.38 *
MM 1597 × MEL 5	−1.66 *	2.48	−0.08	3.35	1.53	−0.16	−1.90	−2.52	−0.65	−0.85	−2.12
MM 1597 × INS2	0.72	2.68	−0.81 **	5.07	1.37	−0.90	−1.17	−2.00	−2.81 ***	−1.05	−1.40
DH 621 × AN-S-26	−1.52 *	0.04	0.02	−1.80	2.20	−1.14	−0.43	−1.83	0.95 *	−1.20	−1.57
DH 621 × H15	0.58	−2.43	−0.21	−3.81	0.51	0.56	0.70	−0.02	0.17	0.42	0.60
DH 621 × A0416	−1.83 **	−1.11	−0.42	−7.14 **	−3.53 **	3.30 ***	3.97 ***	5.14 ***	0.60	1.66	2.58 *
DH 621 × IVIA-371	2.28 ***	−1.97	0.02	−2.21	0.34	−0.26	1.51	0.77	1.33 **	0.60	1.43
DH 621 × ASI-S-1	−0.51	−2.48	0.13	11.36 ***	−0.21	−1.00	−2.02	−1.20	−0.90	0.85	0.54
DH 621 × MEL 1	−1.16	3.37 *	1.09 ***	5.43	1.46	−0.80	−0.97	−1.80	−0.37	0.27	−0.53
DH 621 × MEL 5	1.07	−3.17 *	−0.43	−13.99 ***	−3.50	−0.28	7.42 ***	7.52 ***	0.30	3.52 ***	6.26 ***
DH 621 × INS2	−1.39	0.90	−0.36	7.16 *	3.40 **	−1.08	−2.88 ***	−4.52 ***	−0.56	−3.13 ***	−5.16 ***
AN-S-26 × H15	1.26	0.08	0.24	0.03	4.30 ***	−0.15	−2.10	−4.29 **	0.65	0.14	0.02
AN-S-26 × A0416	1.93 ***	−3.90 **	0.22	0.60	−1.35	1.00	1.12	1.75	−0.42	−1.27	−1.68
AN-S-26 × IVIA-371	0.94	−1.13	−0.92 ***	−7.23 *	1.26	0.07	−0.84	−1.50	1.10 *	−0.82	−0.75
AN-S-26 × ASI-S-1	1.01	4.90 ***	0.56 *	3.14	−3.64 **	2.31 ***	2.10	3.81 *	0.20	−0.05	−0.84
AN-S-26 × MEL 1	1.70 *	−0.82	0.17	−3.77	1.54	−1.82 **	0.00	−0.98	−0.53	1.71 *	3.58 ***
AN-S-26 × MEL 5	−1.05	0.80	0.44	1.35	−0.05	−0.84	0.27	0.18	−0.92	−0.40	−0.70
AN-S-26 × INS2	−1.62 *	−2.76	−0.22	−0.70	2.54 *	−1.37 *	3.08 ***	0.37	0.52	−1.60	−2.20
H15 × A0416	1.06	−2.60	0.02	1.54	0.07	−1.58 *	1.55	1.08	−0.03	1.95 *	3.03 **
H15 × IVIA-371	−0.79	5.97 ***	−0.55	−9.62 ***	−0.51	0.57	−0.02	0.23	−0.96 *	−0.66	−0.91
H15 × ASI-S-1	−1.08	2.73	0.11	−4.72	−0.86	−0.43	0.25	0.70	0.10	−0.35	−0.82
H15 × MEL 1	−1.50 *	1.80	0.48	−0.85	−0.26	0.66	3.27 **	2.56	−0.02	−1.55	−0.81
H15 × MEL 5	−0.86	1.28	0.20	0.15	−0.96	−0.31	−1.28	−0.12	−0.07	−1.70 *	−2.58 *
H15 × INS2	1.12	−3.66 **	−0.46	−5.44	−1.61	−0.70	−1.44	0.20	0.76	9.56 ***	10.28 ***
A0416 × IVIA-371	1.87 **	−0.82	0.55	8.61 **	−1.33	−0.20	−3.37 ***	−0.83	−1.01 *	−0.70	−0.90
A0416 × ASI-S-1	−0.66	−2.26	0.27	−2.15	0.20	−0.50	2.78 *	1.79	0.50	−0.85	−0.57
A0416 × MEL 1	−0.81	0.73	0.07	−3.38	−0.08	−2.18 ***	1.51	1.41	0.13	0.32	0.70
A0416 × MEL 5	0.21	−1.46	−0.23	3.11	−2.97 *	−0.66	4.82 ***	5.54 ***	0.21	0.11	1.00
A0416 × INS2	−1.82 **	−2.35	−0.17	−1.46	−4.64 ***	1.38 *	2.41 *	5.08 ***	−1.70 ***	0.26	−0.42
IVIA-371 × ASI-S-1	−0.77	1.86	−0.25	−4.98	−0.52	−0.32	−2.87 *	−1.27	0.94	−0.27	−0.44
IVIA-371 × MEL 1	−1.76 *	−4.61 ***	−0.03	−6.58 *	−1.94	−0.03	3.96 ***	4.15 **	0.92	−2.03 *	−1.28
IVIA-371 × MEL 5	2.64 ***	3.54 *	0.55	12.25 ***	3.00 *	1.25	−5.16 ***	−5.86 ***	−1.13 *	0.22	−0.57
IVIA-371 × INS2	0.47	2.80	0.51	13.44 ***	4.50 ***	−0.20	−4.34 ***	−6.12 ***	−1.81 ***	−4.76 ***	−6.82 ***
ASI-S-1 × MEL 1	1.12	1.83	−0.79 **	7.80 **	0.68	0.17	−2.20	−2.05	−0.92	−0.64	−0.98
ASI-S-1 × MEL 5	−0.28	2.27	0.09	2.13	0.11	−0.48	−1.30	−0.87	−0.95 *	3.13 ***	3.08 **
ASI-S-1 × INS2	−1.25	0.71	0.31	−3.70	−4.48 ***	1.05	3.60 ***	5.62 ***	−1.27 **	−2.78 ***	−2.60 *
MEL 1 × MEL 5	−1.08	−3.56 *	−0.22	3.14	−2.97 *	0.37	1.70	3.20 *	0.70	0.08	0.77
MEL 1 × INS2	0.78	−1.02	−0.05	−3.40	1.10	0.74	−0.66	−1.28	1.16 *	5.93 ***	5.45 ***
MEL 5 × INS2	−0.98	2.16	−0.10	4.94	2.65 *	0.55	−1.20	−2.92	−1.00 *	−1.22	−2.30 *

***, **, * indicate significant at *p* < 0.001, *p* < 0.01, or *p* < 0.05, respectively.

**Table 5:** Pearson linear correlations for all 13 biochemical traits studied.

	Phenolics	Chlorogenic Acid (mg/g)	Area%	L*_0_	a*_0_	b*_0_	DW_0_	PPO Activity	DB	CD
Dry matter	0.17	0.03	−0.15	−0.22	−0.05	0.03	0.13	0.26 *	0.39 ***	0.44 ***
Total phenolics		0.18	0.00	−0.12	0.02	−0.09	0.01	−0.12	0.04	0.01
Chlorogenic acid (mg/g)			−0.23	−0.13	0.23	−0.03	0.05	0.20	0.00	0.04
Area%				0.52 ***	0.12	−0.49 ***	−0.56 ***	−0.47 ***	−0.30 *	−0.36 **
L*_0_					−0.12	−0.64 ***	−0.89 ***	−0.34 **	−0.47 ***	−0.58 ***
a*_0_						−0.36 **	−0.17	0.13	0.14	0.06
b*_0_							0.92 ***	0.13	0.07	0.26
DW_0_								0.25	0.28 *	0.45 ***
PPO activity									0.43 ***	0.50 ***
DB										0.96 ***

***, **, * indicate significant at *p* < 0.001, *p* < 0.01, or *p* < 0.05, respectively.

**Table 6:** Correlations between genetic distances among parents and hybrid trait values, heterosis (Het), and specific combining ability (SCA).

	All Parents			Only *S. melongena* Parents		
Traits	Trait	*Het*	SCA	Trait	*Het*	SCA
Dry matter	0.125	−0.176	−0.143	−0.125	−0.043	−0.120
Phenolics	0.186	0.143	−0.048	−0.093	0.235	−0.068
Chlorogenic Acid	0.181	−0.137	−0.112	−0.003	0.100	−0.013
Area%	0.001	0.147	0.203	0.224 ^ns^	0.007	0.251
L*_0_	−0.075	0.050	−0.120	0.371 *	−0.171	−0.366 *
a*_0_	0.388 **	−0.113	−0.026	0.404 *	0.039	−0.115
b*_0_	−0.359 *	−0.171	0.043	−0.433 **	−0.030	0.167
DW_0_	−0.203	−0.138	0.085	−0.446 **	0.080	0.286
PPO Activity	0.147	−0.421 **	−0.337 *	−0.139	−0.327	−0.324
DB	0.443 **	0.212	0.129	0.073	0.213	0.113
CD	0.336 *	0.047	0.045	−0.136	0.193	0.105

**, * indicate significant at *p* < 0.01, or *p* < 0.05, respectively.

## Supplementary Materials

Supplementary materials can be found at https://www.mdpi.com/1422-0067/20/12/2990/s1. Table S1: The mean performance of parents and their hybrids for the fruit biochemical traits. Figure S1. Phylogenetic trees constructed based on UPGMA method using SNPs (left) vs. 11 traits studied (right) with tanglegrams.

## Abbreviations

CD	Fruit flesh colour difference
CGA	Chlorogenic acid content
DB	Fruit flesh degree of browning
DW_0_	Degree of whiteness
GCA	General combining ability
GD	Genetic distance
HPLC	High-performance liquid chromatography
PPO	Polyphenol oxidase activity
SCA	Specific combining ability
SNP	Single nucleotide polymorphism
