# Supplementary material for: Genetic Analysis for Fruit Phenolics Content, Flesh Color, and Browning Related Traits in Eggplant (Solanum melongena)
Source: Int J Mol Sci. 2019 Jun 19;20(12):2990. doi: 10.3390/ijms20122990 (PMC6628304; doi:10.3390/ijms20122990)
Supplement: Supplementary file 1 [file ijms-20-02990-s001.zip › Supplementary/Figure S1.docx]

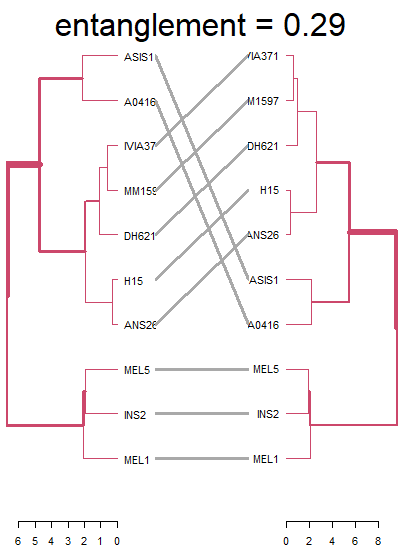


**Figure S1.** Phylogenetic trees constructed based on UPGMA method using SNPs (Left) vs 11 traits studied (Right) with tanglegrams.
